# Joint analysis of structured and semi-structured community science data improves precision of relative abundance but not trends in birds

**DOI:** 10.1038/s41598-022-23603-0

**Published:** 2022-11-24

**Authors:** Alexander R. Schindler, Stephanie A. Cunningham, Toryn L. J. Schafer, Emily A. Sinnott, Sarah J. Clements, Frances M. DiDonato, Alisha R. Mosloff, Clay M. Walters, Amy A. Shipley, Mitch D. Weegman, Qing Zhao

**Affiliations:** 1 Department of Biology, University of Saskatchewan, Saskatoon, SK Canada; 2 School of Natural Resources, University of Missouri, Columbia, MO USA; 3 Department of Environmental Biology, State University of New York College of Environmental Science and Forestry, Syracuse, NY USA; 4 Department of Statistics and Data Science, Cornell University, Ithaca, NY USA; 5 Bird Conservancy of the Rockies, Fort Collins, CO USA

**Keywords:** Ecology, Ecology, Conservation biology, Ecological modelling, Population dynamics

## Abstract

Estimating absolute and relative abundance of wildlife populations is critical to addressing ecological questions and conservation needs, yet obtaining reliable estimates can be challenging because surveys are often limited spatially or temporally. Community science (i.e., citizen science) provides opportunities for semi-structured data collected by the public (e.g., eBird) to improve capacity of relative abundance estimation by complementing structured survey data collected by trained observers (e.g., North American breeding bird survey [BBS]). We developed two state-space models to estimate relative abundance and population trends: one using BBS data and the other jointly analyzing BBS and eBird data. We applied these models to seven bird species with diverse life history characteristics. Joint analysis of eBird and BBS data improved precision of mean and year-specific relative abundance estimates for all species, but the BBS-only model produced more precise trend estimates compared to the joint model for most species. The relative abundance estimates of the joint model were particularly more precise than the BBS-only estimates in areas where species detectability was low resulting from either low BBS survey effort or low abundance. These results suggest that community science data can be a valuable resource for cost-effective improvement in wildlife abundance estimation.

## Introduction

Understanding spatial and temporal variation in wildlife abundance is a fundamental question in population and community ecology. Estimating abundance is challenging, however, as surveys cannot cover all areas and animals cannot be counted with perfect detection. Survey data based on a structured sampling design allows practitioners to quantify observation error and thus make reasonable inference despite imperfect detection, but such surveys are often limited in spatial extent because of financial or logistical constraints. Community science (i.e., citizen science, public participation in scientific research) data collected by volunteer observers has emerged as an essential resource to address such limitations and fill crucial information gaps in monitoring, conservation, and ecological research^1,2^. Community science data can provide information at larger scales not feasible with traditional structured surveys and increase public engagement in conservation^3^, but also has shortcomings such as uneven sampling over time and space, uneven sampling effort per visit, and variability in volunteer abilities to detect different species^4^. Participation in community science programs has increased steadily^3^ along with development of new analytical techniques that increase the utility of data collected for informing conservation efforts (e.g. Refs.^5,6^).

Data integration has increasingly been used to improve estimation and inference capacity through joint analysis of separate but complementary data^7^. Integrated models share parameters between component sub-models to overcome potential parameter estimate biases and missing information present in individual datasets^4^. For example, presence-absence data can be combined with presence-only data for improved models of species distribution^8^. Integrating community science data with other structured data sources could improve insight about ecological patterns and processes^9,10^.

A wealth of bird-specific structured survey data is readily available and accessible to scientists and conservation practitioners. One of the most extensive of these datasets is the North American Breeding Bird Survey (BBS)^11^. BBS is a long-term, international monitoring program of North American bird species conducted by the U.S. Geological Survey and Canadian Wildlife Service that has been running annually during peak bird breeding (May and June) since 1966. BBS data can be used to estimate relative abundance while accounting for observation error because these data follow a standardized protocol^12^. BBS data are commonly used to study population trends and species distributions^13,14^, yet observer availability and restriction to roads may lead to spatially unequal sampling and limited representation of some habitat types and species^15–18^.

Semi-structured data, on the other hand, often cover a larger spatial extent than structured surveys, but generally lack aspects of rigorous protocols^19^ (e.g., trained observers, structured routes, etc.). eBird is one of the largest community science projects in North America. It is maintained by the Cornell Lab of Ornithology and National Audubon Society and allows observers to contribute observations to a publicly available database^20^. As of 2020, 915 million bird sightings had been uploaded to eBird and over 400 peer-reviewed scientific papers had been published making use of these data (https://ebird.org). eBird data have been used to quantify population trends^21^, species distributions^22^, and relative abundance^23^. Yet the semi-structured nature of eBird observations can present challenges to analyzing and interpreting data. Participants preferentially record some species over others (unequal taxonomic sampling), and observations are most common near populated or easy-to-access areas, on weekends, or during migration events (spatially and temporally unequal sampling), and there are often large discrepancies between number of detections and non-detections. However, data filtering practices can substantially reduce these^24,25^.

While it is difficult to estimate relative abundance using eBird alone, both BBS and eBird data can provide strong estimates of population trends^13,14^. Several studies have used eBird and BBS data to quantify species distributions using hierarchical models. Dorazio^26^ developed a species distribution model to jointly analyze planned survey data and opportunistic presence-only data which reduced biases in environmental covariate estimates obtained from models using presence-only data alone. Pacifici et al.^27^ found a joint analysis of eBird and BBS data improved occupancy estimates and predicted species distributions of brown-headed nuthatches (*Sitta pusilla*), compared to analyses using eBird data alone. A joint analysis of relative abundance using BBS and eBird data leveraging strengths of each dataset has the potential to improve the value and precision of range-wide relative abundance and trend estimates^28^, yet such an approach has not been developed or evaluated.

In this study, we developed two models to estimate relative abundance and population trends in 1-degree grid cells across bird species ranges in North America: one using only BBS data (hereafter BBS model) and the other jointly analyzing BBS and eBird data (hereafter joint model). Differences in species life histories or other characteristics (e.g., spatial extent of distributions, density) can affect strength of inference from population models^29^. For example, population trends may be predicted more accurately with eBird and BBS for species with broader spatial and elevational ranges^13^. We evaluated differences in the precision of relative abundance and population trend estimates between BBS-only and joint models within and among bird species in five orders and six families representing a variety of morphologies, life history characteristics, and distributions, likely relating to different detection potential. We expected precision of relative abundance and population trends would be generally improved in joint models relative to BBS-only models. We then examined spatial variation in relative abundance and trend estimates from BBS-only and joint models and anticipated that improvements in precision would be greatest in areas of low relative abundance or at the periphery of a species distribution.

We assessed the relative abundance and population trend estimates of the BBS-only and joint models for a suite of species, as the extent to which migration strategy, habitat preference, and/or population trend influences estimate of relative abundance has not been evaluated: American woodcock (*Scolopax minor*), black-billed cuckoo (*Cocczycus erythropthalmus*), black-throated blue warbler (*Setophaga caerulescens*), Canada goose (*Branta canadensis*), loggerhead shrike (*Lanius ludovicianus*), northern bobwhite (*Colinus virginianus*) and upland sandpiper (*Bartramia longicauda*). American woodcock, black-billed cuckoo, black-throated blue warbler, Canada goose and upland sandpiper are migratory birds^30–34^, loggerhead shrike are partial migrants^35^ and northern bobwhite are non-migratory^36^. These species have diverse habitat preferences including interior forest, forest opening/edge, open grassland, and wetland^30–36^, which may result in variation in detectability. We anticipated that relative abundance estimates for less detectable species (e.g., those rare or cryptic, inhabiting dense forest habitat) would have greatest improvement in precision with additional data relative to more detectable species (e.g., common species, inhabiting open habitat easily seen from a road). We further anticipated that precision in trend estimates would increase for species that are detected less frequently or have greater variability in detection rates^37^. We evaluated precision in relative abundance and trend estimates from BBS-only and joint models within and among species using the coefficient of variation (CV; see “Methods” section).

## Results

**Figure 1:**
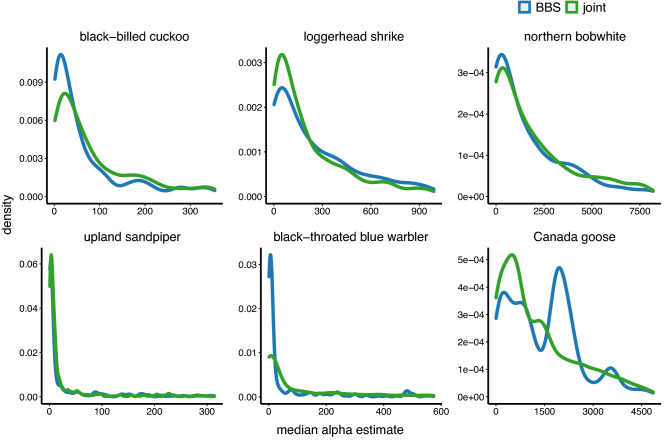
Distribution of per-grid cell relative abundance estimates across respective study areas for BBS only and joint models for black-billed cuckoo, loggerhead shrike, northern bobwhite, upland sandpiper, black-throated blue warbler, and Canada goose. The x-axis represents the range of per-grid cell estimates of mean relative abundance ($\alpha$) from the respective models and the y-axis represents the density of the respective $\alpha$ estimates among all grid cells (i.e., the relative frequency of grid cells with the respective relative abundance).

Estimated mean relative abundance ($\alpha$) per grid cell was similar within species between BBS-only and joint models (Fig. 1). Generally, the relative abundance indices had high frequency of low to moderate relative abundance (i.e., the distribution peaked at low densities of birds; Fig. 1), except for Canada goose, whose BBS abundance peaked at a relatively large abundance per grid cell, but also had moderate frequency of low to moderate relative abundance. The American woodcock BBS and joint models would not converge (i.e., $\widehat{R}$ > 1.2 and chains did not mix). The model converged only when we added an additional data type (i.e., Singing Ground Survey [SGS]).

Mean ($\alpha$) as well as year-specific ($\gamma$) relative abundance estimates for all species were more precise from the joint model relative to the BBS model (i.e., > 50% of grid cells had lower CV from the joint model relative to CV from the BBS model). The proportion of grid cells with lower CV for the joint model relative to the BBS model for mean and year-specific relative abundances were 0.98 and 0.99 for black-billed cuckoo, 0.93 and 0.93 for northern bobwhite, 0.92 and 0.88 for loggerhead shrike, 0.78 and 0.90 for upland sandpiper, 0.64 and 0.79 for black-throated blue warbler, and 0.58 and 0.84 for Canada goose, respectively. Thus, among species, precision across grid cells was least improved in the mean relative abundances of Canada goose and black-throated blue warbler.

**Figure 2:**
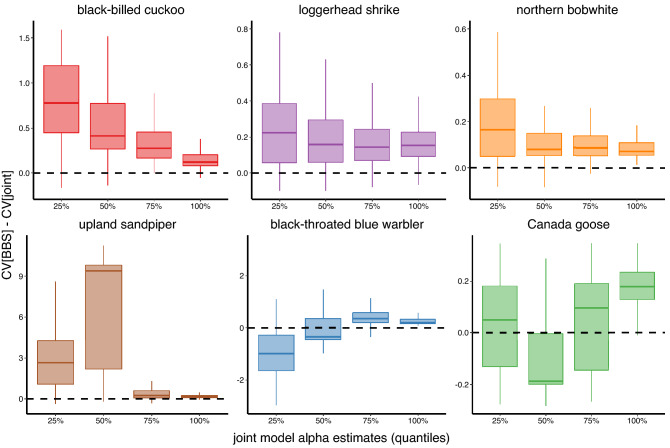
Improvements in precision (i.e., CV[BBS model]–CV[joint model]) of mean relative abundance ($\alpha$) estimates for each grid cell in response to corresponding black-billed cuckoo, loggerhead shrike, northern bobwhite, upland sandpiper, black-throated blue warbler, and Canada goose relative abundances. Relative abundance values on the x-axis represent joint model $\alpha$ estimates and are depicted as 25 percentile ranges. Values above the dashed line indicate that estimates from a joint model were more precise than those from a BBS model, whereas values below that indicate that estimates from a joint model were less precise.

**Figure 3:**
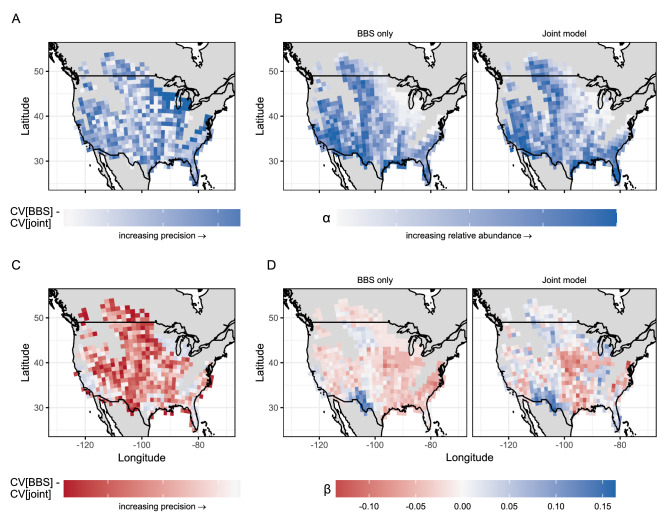
Comparison of precision (i.e., CV[BBS]–CV[joint]); (**A,C**) between BBS-only and joint models for relative abundance (*α*; top row) and trend (*β*; bottom row) of loggerhead shrike. Model-specific estimates of mean relative abundance in are shown in (**B**), and estimates of mean trend in (**D**). In all panels, blue indicates a positive value/increase and red a negative value or decrease, while 0 is set to white.

**Figure 4:**
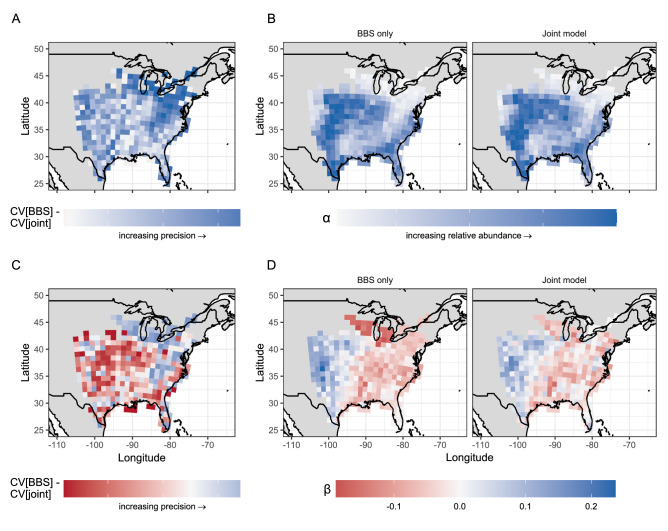
Comparison of precision (i.e., CV[BBS]–CV[joint]); (**A,C**) between BBS-only and joint models for relative abundance (*α*; top row) and trend (*β*; bottom row) of northern bobwhite. Model-specific estimates of mean relative abundance in are shown in (**B**), and estimates of mean trend in (**D**). In all panels, blue indicates a positive value/increase and red a negative value or decrease, while 0 is set to white.

Mean relative abundance CV differences between BBS-only and joint models were greatest (i.e., lowest joint model CVs relative to BBS-only model CVs) for black-billed cuckoo, loggerhead shrike, and northern bobwhite for grid cells with lowest relative abundances and least (i.e., highest joint model CVs relative to BBS-only model CVs) for grid cells with highest relative abundances, as hypothesized. In other words, greatest improvements in precision using the joint model instead of the BBS-only model were realized under lowest estimated mean relative abundances for these species (Figs. 2, 3, 4, Fig. S2). Mean relative abundance CV differences for upland sandpiper also generally followed this pattern, except for grid cells in the 25th to 50th percentile, where gain in precision exceeded that below the 25th percentile (Fig. 2, Fig. S3). Mean relative abundance CV differences for black-throated blue warbler and Canada goose increased with relative abundance whereby greatest improvements in precision between BBS-only and joint models occurred in grid cells with highest relative abundances (Fig. 2, Figs. S4, S5). There were no substantial patterns in the mean relative abundance CV differences for distance to edge of range; precision in relative abundance estimates did not markedly improve in the joint model at greatest distances to edge from species distribution centroid (Fig. S6).

Mean relative abundance CV differences between BBS-only and joint models for black-billed cuckoo, loggerhead shrike, northern bobwhite, and upland sandpiper were greatest in grid cells with fewest BBS routes (Fig. S8). For these species, precision was most improved in the joint model relative to the BBS-only model in grid cells with few BBS routes. However, for black-throated blue warbler and Canada goose, mean relative abundance CV differences between BBS-only and joint were greatest in grid cells with greatest number of BBS routes (Fig. S8). Mean relative abundance CV differences between BBS-only and joint models were greatest in grid cells with fewest eBird checklists for upland sandpiper, but greatest in grid cells with greatest number of eBird checklists for black-throated blue warbler and Canada goose. There were no substantial patterns in mean relative abundance CV improvements with number of eBird checklists for the other species (Fig. S10).

**Figure 5:**
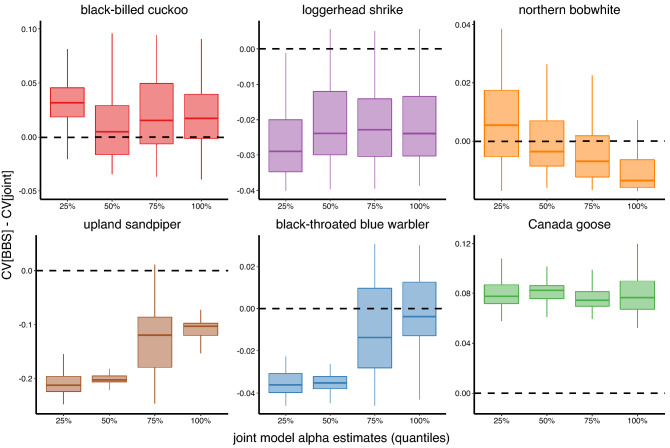
Improvements in precision (i.e., CV[BBS model]–CV[joint model]) of trend ($\beta$) estimates for each grid cell in response to corresponding black-billed cuckoo, loggerhead shrike, northern bobwhite, upland sandpiper, black-throated blue warbler, and Canada goose relative abundances. Relative abundance values on the x-axis represent joint model $\alpha$ estimates and are depicted as 25 percentile ranges. Values above the dashed line indicate that estimates from a joint model were more precise than those from a BBS model, whereas values below that indicate that estimates from a joint model were less precise.

Trend ($\beta$) estimates for black-billed cuckoo and Canada goose were more precise in the joint model relative to the BBS model (proportion of grid cells where joint model CV was lower = 0.68 and 1.00, respectively). Trend estimates for most grid cells for all other species were more precise in the BBS model relative to the joint model. Trend estimates for northern bobwhites were more precise in the joint model relative to the BBS model (i.e., joint model CVs were lower than BBS-only model CVs) in some grid cells with low relative abundance, in the core area of the range (i.e., smallest distance to edge), or with few BBS routes (Figs. 4, 5, Figs. S7, S9). Trend estimates for black-throated blue warbler were more precise in the joint model relative to the BBS model in some grid cells with many eBird checklists (Fig. S11). The number of BBS routes and eBird checklists were strongly correlated only for black-throated blue warbler and Canada goose (i.e., *r* ~ 0.68; Fig. S12).

## Discussion

Information about spatiotemporal variation in abundance is crucial for conservation planning and management of wildlife species. We developed models that jointly analyzed North American Breeding Bird Survey (BBS) and eBird data, using bird species representing a variety of life histories, which estimated spatially explicit indices of mean relative abundance across study years ($\alpha$), indices of year-specific relative abundance ($\gamma$), and trends in relative abundance indices ($\beta$) from 2010 to 2019. Mean and year-specific relative abundance estimates from joint models were more precise than those from BBS models for most grid cells for six of the seven species we studied. In the case of our seventh study species, American woodcock, data integration allowed us to estimate relative abundance and trends when we could not do so with one dataset alone. While BBS and eBird data only allow us to estimate an index of relative abundance and not actual abundance at the grid cell level, such indices are a useful tool for conservation practitioners^38–40^. Overall, our modeling framework will allow conservation practitioners to integrate count data collected on different spatiotemporal scales and identify areas that have averaged high densities of a species over time (i.e., high $\alpha$ values), high density areas in a particular year (i.e., high $\gamma$ values), and areas where relative abundance is increasing or decreasing (i.e., positive or negative $\beta$ values, respectively).

As anticipated, CV difference in mean relative abundance was greater when relative abundance was lower, and greatest improvement in CV was generally associated with fewer BBS routes for black-billed cuckoo, loggerhead shrike, northern bobwhite and upland sandpiper. Thus, relative abundance estimates were more precise after adding eBird data when there were fewer detections resulting from either low BBS survey effort or low relative abundance of these species. However, we found the opposite patterns in CV difference in mean relative abundance for black-throated blue warbler and Canada goose (i.e., greatest CV difference occurred at greatest mean relative abundance). For Canada goose, we believe this is a result of limited BBS and eBird data in the northern part of the species range (i.e., BCRs 1–7; Supplementary Information Fig. S13), which contain large areas of temperate or boreal forest, or tundra, with low densities of people. Such conditions impede access by both community scientists and BBS observers^41,42^. We therefore recommend caution when interpreting relative abundance and trend estimates in these areas where survey data are limited. In the case of black-throated blue warbler, the lack of improvement in precision when integrating several data types may be due to black-throated blue warbler habitat use, which may influence detectability in eBird observations and BBS surveys. Black-throated blue warblers are most likely to occur in unfragmented tracts of forest of at least 100 ha and therefore less likely to be detected than species occupying open, more accessible areas regardless of survey type^43–45^. Because the species is both area-dependent and occupies habitats with dense forests and shrubs during the breeding season^32^, it is likely that roadside BBS surveys are less likely to detect it than other species in our study, which occupy open (loggerhead shrike, northern bobwhite, upland sandpiper, Canada goose^33–36^) or forest edge (black-billed cuckoo^31^) habitats. Adding eBird data may only improve precision for areas in which area-dependent species are abundant enough to be easily detected by observers.

Life history differences likely further affected improvements between joint and BBS-only models. Precision in black-billed cuckoo abundance was most improved in joint vs. BBS-only models, while Canada goose abundance estimates were least improved. Black-billed cuckoos are sit-and-wait predators and are generally patchily distributed, making them difficult to detect with traditional point-count surveys^31,46^. In contrast, Canada geese favor open area habitats and are easily detected^33^. Integrating eBird data may therefore be especially beneficial for less detectible (e.g., less vocal or conspicuous) species. However, we acknowledge our seven focal species represent a small sample size and encourage further research to examine how life history differences affect improvements in abundance estimates from data integration. The American woodcock models required a third dataset, the Singing-Ground Survey, to converge. American woodcocks are most detectible in the evening when males exhibit courtship behavior^30^, and detection rates of American woodcocks during the BBS and other standard morning point-count surveys are generally low^47^. While we expected integrating eBird data in American woodcock models would improve parameter estimation, the number of American woodcock detections in eBird data may not have been sufficient without additional data from the Singing Ground Survey, which was designed to maximize detection probability of this species.

Contrary to our hypotheses, our trend estimates were more precise for BBS models compared to models with multiple data types for all species except black-billed cuckoo and Canada goose. In some cases, the joint model estimates indicated an increasing trend where the BBS-only model indicated a declining trend (e.g. Fig. 3, Figs. S2–S4). To illustrate our data integration approach, we structured our models to estimate a simple linear trend in abundance over our study period. Although we used a relatively short time series of data (10 years), we anticipate abundance of many of these species may not have increased or decreased linearly over time. In cases where abundance did not change linearly (e.g., increased for a few years before decreasing, decreased for a few years before decreasing less severely, etc.), increased precision in our relative abundance estimates may have increased uncertainty in our linear trend estimate (i.e., we may have been less likely to detect a linear trend where a linear trend did not exist). In such cases, our BBS model trend estimates may have been overly precise. A modeling framework with the flexibility to estimate nonlinear trends may be more appropriate and yield more accurate trend estimates. We encourage more research to evaluate the tradeoffs between increased precision in abundance and decreased precision in trend estimates.

We anticipated improvements in precision of trend estimates would be greatest in areas of low relative abundance (i.e., adding more data would improve precision in estimates where abundance was low, and birds were therefore less detectible). However, the relationship between trend precision and relative abundance was variable between species. For example, we observed increased precision in mean trend estimates for black-billed cuckoo along the western edge of their range, which had lower estimates for relative abundance, whereas the joint model did not improve precision of trend estimates for some of the grid cells with the highest estimates of relative abundance (in the northern part of the species’ range; Fig. S2). However, in black-throated blue warblers, the areas of greatest relative abundance appeared to have the highest precision in trend estimates (Fig. S4).

Theoretical and empirical work has indicated that abundance of species decreases with increasing distance from center of range, or more accurately, center of niche^48,49^. Therefore, we expected lower relative abundances of birds at the edge of their ranges, and that precision of our joint model would be greater in grid cells on range peripheries. However, we did not find such patterns in any species we examined, and there was little difference in precision between cells at the periphery of range and those in the center (Fig. S6). We anticipate that this may be due, in part, to our calculation of the geographic center rather than niche center, as well as the coarse resolution (12,321 km^2^) of grid cells^50^ and species’ range definitions (i.e., including whole BCRs if any portion of the BCR contained detections of the species), which might not capture sharp declines in relative abundance if they occur over a relatively small distance^51^. Further, strength of relationship may vary by species, according to traits such as body size, niche-breadth, and distribution of species relative to their available niche^48,50^. We suggest practitioners further explore these finer scale applications of our approach to more deeply understand how precision may be improved at range edges.

Locations of surveys and checklists in the BBS and eBird datasets, respectively, are non-random and limited to road proximity (in the case of BBS surveys) and human activity (in the case of eBird surveys), which can lead to biased abundance and trend estimates^52^. We incorporated an observer-by-route error term (${\varepsilon }_{j,s}^{[BBS]}$) in our BBS observation model in part to account for correlation among data collected from the same survey route (i.e., account for routes that were consistently in better or worse habitat). Additionally, we incorporated several data filtering steps in our eBird data set to help reduce biased abundance and trend estimates associated with increased survey effort in areas with higher human activity. However, additional considerations may be needed if researchers are looking to understand population-habitat relationships^53^.

Data integration in ecology has become common over the last 20 years as statistical theory, programming, and computing power have dramatically improved^9^. There is a growing body of literature leveraging multiple data streams to estimate species distribution, relative abundance, and population trends, including using BBS and eBird data^4,54,55^. Pacifici et al.^27^ and Robinson et al.^56^ showed that models with jointly analyzed data allowed for greater inference about species distributions compared to those from single dataset models. Our study provides an important example of estimating relative abundance using data collected at different spatial scales, under different observation protocols, across a gradient of bird life histories. Improvement in precision of abundance estimates provides a promising solution to traditional limitations to conservation investment, where practitioners have been limited to investing resources in places where structured surveys are available. Integrating data streams also allows for cost-effective study design, as researchers do not need to invest in only structured surveys and instead can rely on non-structured data for additional information.

We anticipate our model framework will have widespread utility, as practitioners can adjust our framework to integrate other structured datasets with publicly available, semi-structured data. Our model uses a scaling intercept and additional effort variables to scale the unstructured dataset to the scale of the structured one (informed by the known effective area of the structured survey). In many cases, practitioners may wish to integrate multiple unstructured surveys where the effective survey area is not explicitly known. While such a task is possible with our modeling structure, practitioners would need enough information about the surveys to filter for quality data (e.g., subsample data based on spatial location, temporal attributes, large differences in survey effort, etc.) and control for differences in survey effort (e.g., time of day, distance covered, time duration, etc.), thus imposing some structure on the survey data. Practitioners could then either scale one survey to another in the model or assume that, after controlling for differences in survey effort, each survey informs relative abundance on the same scale.

Integrating data from structured and semi-structured data comes with challenges. The level of model complexity required to properly integrate multiple data types and scale the data so they are comparable may be difficult for many practitioners to implement. Our modeling approach is quite intensive and may require a large amount of data for all parameters to properly converge, particularly when estimating abundance and trends in areas with low densities of study species. Additionally, community science data often requires many filtering steps to impose a “semi-structure” to ensure quality of data, which requires additional work for practitioners. Finally, while we included survey information to account for variation in detection among surveys (i.e., observer, route, survey effort), neither BBS nor eBird surveys were designed to explicitly quantify detection probabilities^57,58^, limiting inference to relative abundance.

We encourage researchers to integrate community science data with structured surveys to improve estimates of abundance, particularly when doing so substantially increases the number of detections (e.g., areas with low densities of the species of interest or low structured survey effort, or when life history traits make a species difficult to detect). Contributions from community scientists to biological monitoring programs have the potential to increase precision of parameter estimates in a cost-effective^1^ way, and we believe these benefits outweigh challenges of incorporating opportunistic or semi-structured datasets in statistical models.

## Methods

### Data acquisition and preparation

#### Structured datasets

We used structured North American Breeding Bird Survey (BBS) data, which is conducted annually over > 2500 routes across the United States and Canada^11,12^ during the peak of the breeding season (May and June). BBS routes were approximately 40 km long with 50 stops spaced 0.8 km apart. At each stop a 3-min point count was conducted, where all species seen or heard were recorded^12^. We downloaded the entire dataset, 1966–2019, to identify each observer’s first year and account for differences in survey experience. We created a binary variable for the observers’ first year, with 1 indicating the first year they provided data, and 0 indicating all subsequent years. We then subset the data to years 2010–2019 to align with available community science data. We zero-filled BBS data by adding zeros for each species on routes in which birds were not detected in each year.

#### Semi-structured dataset

We used the eBird Basic Dataset as a semi-structured dataset. We used checklists within the US and Canada during June and July from 2010 to 2019. Data were filtered to impose structure on the observation process and minimize effects of unequal spatial and temporal sampling using the auk package in program R^24,25,56,59,60^. Data were filtered to only include complete checklists where observers recorded counts of all species detected to reduce effects of preferential species reporting^61^. We also filtered data based on observer effort to only include checklists < 5 h in duration, < 5 km in distance travelled, and checklists with < 10 observers^24^.

Semi-structured community science data are generally sampled unevenly across space and time. Observers tend to collect data more frequently in accessible locations close to where they live or in areas with high species diversity^25^. Therefore, populated areas and protected areas are data rich, while expansive rural, private lands are relatively data poor. Additionally, the number of checklists submitted to eBird has increased over time. To improve spatial and temporal representation within each 1-degree grid cell block and reduce the density of checklists within populated or protected areas, we randomly sampled Canada goose data to include one detection and one non-detection checklist for each cell of a 5-km hexagonal grid in each week of June and July because of their high frequency of detections in submitted checklists^24^. The resulting Canada goose data set consisted of 18% detections and 82% non-detections. American woodcock, black-billed cuckoo, black-throated blue warbler, loggerhead shrike, northern bobwhite and upland sandpiper were detected in < 25% of checklists submitted; there was a high proportion of non-detections compared to detections causing class imbalance. For rare species and highly imbalanced datasets, inference on species distributions may be impeded by the volume of non-detections^62^. We corrected for this class imbalance by retaining all checklists with detections and under-sampling the majority class by spatiotemporally subsampling non-detection checklists only^62^ (i.e., retaining all checklists with detections but only one non-detection checklist for each cell of the 5-km hexagonal grid per week). Resulting datasets consisted of 1–8% detections and 92–99% non-detections.

Each eBird checklist also contained survey information to accompany the associated counts. We used survey start time, duration (in minutes), distance traveled (in km), survey type (stationary or traveling), number of observers, and a unique observer ID number for each observer to account for differences among survey data. We expected our study species to be most active and easily detected in early morning and evening. To capture activity, we converted survey start times to local solar time of day (in seconds) and calculated two continuous covariates with a 24-h period by $\text{cos}\left(\frac{2\pi \left(\text{seconds}\right)}{86400}\right)$ and $\text{sin}\left(\frac{2\pi \left(\text{seconds}\right)}{86400}\right)$, referred to as cos(time) and sin(time). Thus, cos(time) represented night (high values) and day (low values), and sin(time) represented the first half of the day (high values) and the second half of the day (low values)^63^.

### Reference grid

We overlayed a grid of 111-km × 111-km (approximately 1° latitude × 1° longitude near the equator) grid cells using Albers Equal Area projection across Canada and the US to estimate site-specific species relative abundance and trends. We assigned BBS routes and eBird observations to cells based on route starting points and checklist locations. We used the center points of grid cells to assign each to one of the Bird Conservation Regions (BCRs) from the North American Bird Conservation Initiative^64^. We defined species ranges as all BCRs that contained grid cells where the species was observed in any dataset during our study period after removing observations of likely vagrants. We calculated the Euclidean distance of each grid cell centroid to the nearest edge of these defined ranges.

### Model description

For each species, we developed two models to estimate the spatiotemporal variation of species relative abundance. The first model utilized BBS data only and the second model jointly analyzed BBS and eBird data. These models were developed under a hierarchical Bayesian framework, so they shared the same process model but had different observation or data models^65^.

#### Process model

The process model described the trends of relative abundance by expressing relative abundance as a linear function of time (year). More specifically, we assumed that

1 $${\mu }_{i,t}^{\left[\gamma \right]}={\alpha }_{i[k]}^{\left[\gamma \right]}+{\beta }_{i[k]}^{\left[\gamma \right]}\times {YEAR}_{t},$$

and

2 $$\log\left({\gamma }_{i,t}\right) \sim N\left({\mu }_{i,t}^{\left[\gamma \right]}, {\sigma }^{\left[\gamma \right]}\right),$$

in which ${\gamma }_{i,t}$ was year-specific relative abundance for grid cell *i* and year *t*, ${\mu }_{i,t}^{\left[\gamma \right]}$ was the mean of $\log\left({\gamma }_{i,t}\right)$, ${\alpha }_{i[k]}^{\left[\gamma \right]}$ and ${\beta }_{i[k]}^{\left[\gamma \right]}$ were grid cell-specific intercept and slope, respectively, for BCR *k*. The intercept and slope parameters, ${\alpha }_{i[k]}^{\left[\gamma \right]}$ and ${\beta }_{i[k]}^{\left[\gamma \right]}$, were random deviations from a mean intercept and slope for BCR *k*. For example, we had for the slope parameter (${\beta }_{i[k]}^{\left[\gamma \right]}$)

3 $${\beta }_{i[k]}^{\left[\gamma \right]}\sim \text{N}\left({\mu }_{\left[k\right]}^{\left[\beta \right]}, {\sigma }^{\left[\beta \right]}\right), {\mu }_{\left[k\right]}^{\left[\beta \right]}\sim \text{N}\left({{\mu }^{[\beta ]},\uptau }^{\left[\beta \right]}\right),$$

in which ${\mu }_{\left[k\right]}^{\left[\beta \right]}$ is the BCR-specific mean of the slope parameters (${\beta }_{i[k]}^{\left[\gamma \right]}$) for BCR *k* and ${\mu }^{[\beta ]}$ was the grand mean of ${\beta }_{i[k]}^{\left[\gamma \right]}$ for all *i* and *k*. The priors for the mean parameters were assumed to be normally distributed with mean 0 and standard deviation 10 while the priors for the variance parameters were assumed to come from an inverse gamma distribution with shape and rate parameters 0.01. In this way we allowed the intercept and slope parameters to vary across any grid cell, but also assumed that grid cells within the same BCR have more similar values than grid cells in different BCRs^66^. This allowed us to additionally estimate relative abundance and relative abundance trends in grid cells without any count data based on these relationships among grid cells in the same BCR. We used the same model for the grid cell-specific intercepts (${\alpha }_{i[k]}^{\left[\gamma \right]}$) with an intercept grand mean ${\mu }^{[\alpha ]}$, BCR-specific mean ${\mu }_{\left[k\right]}^{\left[\alpha \right]}$, and variance parameters ${\sigma }^{\left[\alpha \right]}$ and ${\uptau }^{\left[\alpha \right]}$, assuming the same priors.

Note that we centered ${YEAR}_{t}$ so that the middle year (i.e., between 2014 and 2015 for our 2010–2019 study period) was 0. Thus $exp\left({\alpha }_{i[k]}^{\left[\gamma \right]}\right)$ was the mean relative abundance for grid cell *i* across the study period, and ${\beta }_{i[k]}^{\left[\gamma \right]}$ the grid cell-specific trend of log relative abundance during the study period. We used Eq. (2) to add additional Gaussian noise to our mean log relative abundance estimates, ${\mu }_{i,t}^{\left[\gamma \right]}$, to account for overdispersion in year-specific relative abundance, ${\gamma }_{i,t}$, in grid cell *i* in year *t*. We assumed the variance parameter ${\sigma }^{[\gamma ]}$ came from an inverse gamma distribution with shape and rate parameters 0.01.

#### Observation model (BBS model)

We hierarchically linked year-specific relative abundance ($\gamma$), and by extension, mean relative abundance ($\alpha$) and relative abundance trends ($\beta$), from the process model to BBS count data (${y}^{[BBS]}$). Because BBS data contained a larger proportion of non-detections (0 counts) than predicted with a Poisson distribution (as determined by posterior predictive checks), we assumed that data were generated from a zero-inflated Poisson process such that

4 $${y}_{j,s,i,t}^{[BBS]}\sim Poisson\left[{\uplambda }_{j,s,i,t}^{\left[BBS\right]}\times \left(1-{z}_{j,s,i,t}^{[BBS]}\right)\right],$$

in which ${y}_{j,s,i,t}^{[BBS]}$ was the BBS count for route *j* (sum of counts across all 50 stops) surveyed by observer *s* in grid cell *i* and year *t*, ${\uplambda }_{j,s,i,t}^{\left[BBS\right]}$ was the expectation of count data ${y}_{j,s,i,t}^{[BBS]}$, and ${z}_{j,s,i,t}^{[BBS]}$ was a Bernoulli random variable with probability ${\omega }^{[BBS]}$, where ${\omega }^{[BBS]} \sim U(0, 1)$.

To achieve grid cell-level inference, we offset the grid cell relative abundance by the effective area surveyed by BBS routes to scale the grid cell relative abundance to BBS route-level relative abundance. More specifically, we assumed

5 $$\text{log}\left({\uplambda }_{j,s,i,t}^{[BBS]}\right)={\text{log}}\left({\gamma }_{i,t}\times (25/\text{12,321})\right)+{\varepsilon }_{j,s}^{[BBS]}+{\eta }^{[BBS]}\times {I}_{j,s,t}^{[BBS]},$$

where $25/\text{12,321}$ was the effective area surveyed by a BBS route (~ 25 km^2^; 50 stops each with a 400 m survey radius^12^) divided by the area of a single grid cell (12,321 km^2^). To account for detection error related to observer experience, we included ${\varepsilon }_{j,s}^{[BBS]}$ as a route- and observer-specific error term, and ${\eta }^{[BBS]}$ as an additional term of observation error if route *j* was surveyed by a first-time observer *s*^12,67^ (indicated by ${I}_{j,s,t}^{[BBS]}$). We assumed ${\varepsilon }_{j,s}^{[BBS]}$ was normally distributed with mean 0 and variance ${\sigma }^{[BBS]}$, where ${\sigma }^{[BBS]}$ came from an inverse gamma distribution with shape and rate parameters 0.01. We assumed ${\eta }^{[BBS]}$ was normally distributed with a mean 0 and standard deviation 10.

#### Observation model (joint model)

In the joint model, we again linked year-specific relative abundance ($\gamma$) from the process model to BBS count data using Eqs. (4) and (5). Similarly, we hierarchically linked year-specific relative abundance to eBird count data (${y}^{[eBird]}$). Thus, both datasets informed year-specific relative abundance ($\gamma$), mean relative abundance ($\alpha$) and relative abundance trend ($\beta$) estimates. We again used a zero-inflated Poisson distribution such that

6 $${y}_{j,s,i,t}^{[eBird]}\sim Poisson\left[{\uplambda }_{j,s,i,t}^{[eBird]}\times \left(1-{z}_{j,s,i,t}^{[eBird]}\right)\right],$$

in which ${y}_{j,s,i,t}^{[eBird]}$ was the eBird count for checklist *j* surveyed by observer *s* in grid cell *i* and year *t*, ${\uplambda }_{j,s,i,t}^{[eBird]}$ was the expected count, and ${z}_{j,s,i,t}^{[eBird]}$ was a Bernoulli random variable with probability ${\omega }^{[eBird]}$, where ${\omega }^{[eBird]} \sim U(0, 1)$.

For eBird data, we again considered the variation among checklists. Instead of observer experience, we considered additional survey effort variables that potentially influenced the distribution of counts. We assumed

7 $$\begin{aligned}\text{log}\left({\uplambda }_{j,s,i,t}^{[eBird]}\right)& =log\left({\gamma }_{i,t}\right)+{\beta }_{0}^{[eBird]}+{\beta }_{1}^{[eBird]}\times {TYPE}_{j,s,i,t}+{\beta }_{2}^{[eBird]}\times {DIST}_{j,s,i,t}+ {\beta }_{3}^{[eBird]}\times {COSTIME}_{j,s,i,t}\\& \quad+\, {{\beta }_{4}^{[eBird]}\times {SINTIME}_{j,s,i,t}+\beta }_{5}^{[eBird]}\times {DURA}_{j,s,i,t}+{ \beta }_{6}^{[eBird]}\times {NOOB}_{j,s,i,t}+{\varepsilon }_{s}^{[eBird]},\end{aligned}$$

in which ${TYPE}_{j,s,i,t}$ was survey type (i.e., 1 for point count and 0 for transect), ${DIST}_{j,s,i,t}$ was survey distance, ${COSTIME}_{j,s,i,t}$ and ${SINTIME}_{j,s,i,t}$ were cos(time) and sin(time), respectively, of start times of the survey, ${DURA}_{j,s,i,t}$ was the survey duration, and ${NOOB}_{j,s,i,t}$ was the number of observers. We included ${\varepsilon }_{s}^{[eBird]}$ as an observer-specific error term to account for correlation among counts from the same observer. We were unable to calculate the effective area surveyed for individual eBird checklists. Instead, we included ${\beta }_{0}^{[eBird]}$, an additional scaling intercept to relate eBird relative abundance estimates from the checklist level to the grid cell level. We assumed ${\beta }_{0-6}^{[eBird]}$ were normally distributed with mean 0 and standard deviation 10. We assumed ${\varepsilon }_{s}^{[eBird]}$ was normally distributed with mean 0 and variance ${\sigma }^{[eBird]}$, where ${\sigma }^{[eBird]}$ came from an inverse gamma distribution with shape and rate parameters 0.01.

#### Additional considerations to improve model convergence

We further altered American woodcock and Canada goose joint models to improve model convergence. The BBS and eBird datasets contained fewer detections of American woodcocks than in other species; thus, we predicted that model convergence issues in the American woodcock models were due to lack of sufficient data across BBS and eBird datasets. To increase our data sample sizes, we used data from the American woodcock Singing Ground Survey (SGS) as a third data source for American woodcock models (see Supplementary Information). The American woodcock SGS is specifically designed for detecting this cryptic species, so detectability is likely higher during this survey compared to surveys not specifically designed for American woodcocks. We developed another model to jointly analyze BBS, eBird, and SGS datasets (see Supplementary Information). This model used the same process model, BBS observation model, and eBird observation model described in Eqs. (1)–(7).

Canada geese frequently form large flocks (often 100 s or 1000 s of individuals), a unique ecological trait among our study species. Thus, both BBS and eBird Canada goose data contained uniquely high counts among our data (max counts of 2900 and 3000 Canada geese in BBS and eBird data, respectively). We predicted that convergence issues in the Canada goose joint model were due to overdispersion resulting from uniquely high counts, especially in the eBird data where these counts were more frequent than in the BBS data. To account for this overdispersion, we added additional Gaussian noise to ${\uplambda }_{j,s,i,t}^{[eBird]}$ in Eq. (7) (see Supplementary Information).

### Model implementation

We used Markov Chain Monte Carlo and a Gibbs sampler in JAGS^68^ using the jagsUI package^69^ in program R^61^. We ran at minimum 9000 iterations, discarded at minimum the first 5000 samples as burn-in, and retained at least 4000 samples with no thinning from 3 chains (exact number of iterations needed to achieve convergence varied among models). We evaluated convergence using the Gelman–Rubin statistic^70^ ($\widehat{R}$ < 1.2) and visual assessments of traceplots. We used a modified CV calculation to assess improvements in precision for $\alpha$, $\gamma$, and $\beta$ (i.e., mean relative abundance, year-specific relative abundance, and trend estimates, respectively) parameters between BBS and joint models. For $\alpha$ and $\gamma$ parameters,

8 $$\begin{array}{l}{CV}^{\left[\alpha \right]}=\left({\widehat{\alpha }}_{\left(0.975\right)}-{\widehat{\alpha }}_{\left(0.025\right)}\right)/\left|{\widehat{\alpha }}_{\left(0.5\right)}\right|\\ {CV}^{[\gamma ]}=\left({\widehat{\gamma }}_{(0.975)}-{\widehat{\gamma }}_{(0.025)}\right)/\left|{\widehat{\gamma }}_{(0.5)}\right|\end{array},$$

and for $$\beta$$

9 $${CV}^{[\beta ]}=\left({\widehat{\beta }}_{(0.975)}-{\widehat{\beta }}_{(0.025)}\right),$$

where ${\widehat{\alpha }}_{(p)}$, ${\widehat{\gamma }}_{(p)}$, and ${\widehat{\beta }}_{(p)}$ are the *p*th quantiles of the posterior distribution for $\alpha$, $\gamma$, and $\beta$, respectively. Smaller CVs indicated more precise estimates of comparable parameters. We calculated CVs of each parameter for each grid cell. We subtracted the joint model CVs for each parameter for each grid cell from corresponding BBS-only model CVs to evaluate the magnitude of improvement in precision for each grid cell and evaluated overall improvements for each parameter by calculating the proportion of grid cells for which joint model CVs < corresponding BBS-only model CVs.

## Supplementary Information

Supplementary Information 1.

Supplementary Figure S2.

Supplementary Figure S3.

Supplementary Figure S4.

Supplementary Figure S5.

Supplementary Figure S6.

Supplementary Figure S7.

Supplementary Figure S8.

Supplementary Figure S9.

Supplementary Figure S10.

Supplementary Figure S11.

Supplementary Figure S12.

Supplementary Figure S13.

Supplementary Information 2.

Supplementary Information 3.

## Data Availability

All data used in this manuscript are publicly available through eBird (Cornell Lab of Ornithology, National Audubon Society), North American Breeding Bird Survey (United States Geological Survey, Environment & Climate Change Canada), and American Woodcock Singing Ground Survey (United States Fish & Wildlife Service).
