# Supplementary material for: Joint analysis of structured and semi-structured community science data improves precision of relative abundance but not trends in birds
Source: Sci Rep. 2022 Nov 24;12:20289. doi: 10.1038/s41598-022-23603-0 (PMC9700822; doi:10.1038/s41598-022-23603-0)
Supplement: Supplementary file 1 — Supplementary Information 1. [file 41598_2022_23603_MOESM1_ESM.docx]

**Supplementary Information**

**Joint analysis of structured and semi-structured community science data improves precision of relative abundance but not trends in birds**

Alexander R. Schindler^1,2^, Stephanie A. Cunningham^3^, Toryn L. J. Schafer^4^, Emily A. Sinnott^2^, Sarah J. Clements^2^, Frances M. DiDonato^2^, Alisha R. Mosloff^2^, Clay M. Walters^1,2^, Amy A. Shipley^2^, Mitch D. Weegman^1,2^, Qing Zhao^2,5^

*^1^Department of Biology, University of Saskatchewan, Saskatoon, SK, Canada*

*^2^School of Natural Resources, University of Missouri, Columbia, MO, USA*

*^3^Department of Environmental Biology, State University of New York College of Environmental Science and Forestry, Syracuse, NY, USA*

*^4^Department of Statistics and Data Science, Cornell University, Ithaca, NY, USA*

*^5^Bird Conservancy of the Rockies, Fort Collins, CO, USA*

*American Woodcock Singing Ground Survey (SGS) Data*

We used data from the Singing Ground Survey (SGS) as a third data source for the American woodcock models. The SGS occurs annually along roads during peak American woodcock breeding activity in spring^1,2^. Routes are 5.4 km long with 10 stops and the entire survey takes ≤38 minutes^1,3^. At each stop, observers record the number of American woodcocks heard within 2 minutes^3^, and we assumed that all birds heard were within 309 m of the observer^4^. Survey periods vary slightly according to latitude to match timing of courtship behavior across the species’ range^2^. The SGS has occurred annually since 1968, though only approximately two-thirds of the 1,500 routes are surveyed each year; the remainder are given the status of “constant zero^2^”. Routes are designated as “constant zero” if there are no detections in two consecutive years and are only surveyed again after five years^2^. An observer’s first year was designated following the same procedure used for BBS data. We did not zero-fill these data, because the routes presumed to be zero are not surveyed.

When assigning routes to 1° grid cells used for analysis, we assigned SGS routes to the grid cell that encompassed center point of the county in which the route took place. Coordinates were unavailable for the SGS survey starting locations.

*Modifications to American woodcock and Canada goose models to improve convergence*

We linked SGS data for American woodcock with abundance using a zero-inflated Poisson distribution such that

$y_{j,s,i,t}^{[SGS]}\sim Poisson\left[ \lambda_{j,s,i,t}^{\left[ SGS \right]}\times\left( 1-z_{j,s,i,t}^{[SGS]} \right) \right]$

in which $y_{j,s,i,t}^{[SGS]}$ was the SGS count for route *j* surveyed by observer *s* in grid cell *i* and year *t*, $\gamma_{j,s,i,t}^{\left[ SGS \right]}$ was the expected count, and $z_{j,s,i,t}^{[SGS]}$ was a Bernoulli random variable with probability $\omega^{[SGS]}$, where $\omega^{[SGS]} \sim U(0, 1)$.

To achieve grid cell-level inference, we similarly used estimates of the effective area surveyed by SGS routes to convert estimates of individuals per route to individuals per grid cell. We assumed

$log\left( \lambda_{j,s,i,t}^{[SGS]} \right)=log\left( \gamma_{i,t}\times(3/{12,321}) \right)+\varepsilon_{j,s}^{[SGS]}+\eta^{[SGS]}\times I_{j,s,t}^{[SGS]}$

where 3/12,321 was the effective area surveyed by an SGS route (~3 km^2^; 10 stops each with a 309 m survey radius^4^) divided by the area of a single grid cell (12,321 km^2^). To account for error related to the observer’s experience, we included $\varepsilon_{j,s}^{[SGS]}$ as a route- and observer-specific error term, and $\eta^{[SGS]}$ as an additional term of observation error if route *j* was surveyed by a first-time observer *s* (indicated by $I_{j,s,t}^{[SGS]}$)^5,6^. We assumed the same priors as in the BBS observation model for corresponding parameters.

To account for overdispersion predicted in the eBird count data for Canada goose, we added additional Gaussian noise to $\lambda_{j,s,i,t}^{[eBird]}$ in equation 7, such that

$log\left( \lambda_{j,s,i,t}^{[eBird]} \right) \sim N(\mu_{j,s,i,t}^{[eBird]}, \sigma^{[\lambda]})$

where $\mu_{j,s,i,t}^{[eBird]}$ is the mean expectation of the log count and $\sigma^{[\lambda]}$ is an additional Gaussian noise parameter which we assumed came from an inverse gamma distribution with shape and rate parameters 0.01. Thus, we accounted for variation among checklists by assuming

$log\left( \mu_{j,s,i,t}^{[eBird]} \right)=log\left( \gamma_{i,t} \right)+\beta_{0}^{[eBird]}+\beta_{1}^{[eBird]}\times{TYPE}_{j,s,i,t}+\beta_{2}^{[eBird]}\times{DIST}_{j,s,i,t}+ \beta_{3}^{[eBird]}\times{COSTIME}_{j,s,i,t}+{\beta_{4}^{[eBird]}\times{SINTIME}_{j,s,i,t}+\beta}_{5}^{[eBird]}\times{DURA}_{j,s,i,t}+\beta_{6}^{[eBird]}\times{NOOB}_{j,s,i,t}+\varepsilon_{s}^{[eBird]}$

in whichthe same effort variables as equation 7 influenced the distribution of counts.

*References*

1. Rau, R. D., Cooper, T. R. & Nelson, M. R. American Woodcock Signing-ground Survey: The logistical challenges associated with route consistency through time. *Proc. Am. Woodcock Symp.* **11**, 217-226 (2017).
2. Seamans, M. & Rau, R. D. American Woodcock status. *Proc. Am. Woodcock Symp.* **11**, 9-17 (2017).
3. U.S. Fish and Wildlife Service. American Woodcock Singing-ground Survey training tools document. <https://migbirdapps.fws.gov/woodcock/trainingtooldocs.htm>; Accessed 21 January 2021 (2017).
4. Bergh, S. M. & Andersen, D. E. Estimating density and effective area surveyed for American Woodcock. *Proc. Am. Woodcock Symp.* **11**, 193-199 (2017).
5. Sauer, J. R. & Link, W. A. Analysis of the North American Breeding Bird Survey using hierarchical models. *Auk* **128**, 87–98 (2011).
6. Bled, F., Sauer, J., Pardieck, K., Doherty, P. & Royle, J. A. Modeling trends from North American breeding bird survey data: A spatially explicit approach. *PLoS ONE* **8**, e81867; <https://doi.org/10.1371/journal.pone.0081867> (2013).
7. Bird Studies Canada & NABCI. Bird Conservation Regions. <https://www.birdscanada.org/bird-science/nabci-bird-conservation-regions/> (2014).

*Figures and Tables*

| species | $\beta_{0}^{[eBird]}$ | | |
| --- | --- | --- | --- |
|  | LCI | median | UCI |
| AMWO | -5.471 | -4.866 | -4.225 |
| BBCU | -9.015 | -8.874 | -8.742 |
| BTBW | -9.016 | -8.923 | -8.766 |
| CAGO | -6.304 | -6.226 | -6.153 |
| LOSH | -8.366 | -8.238 | -8.133 |
| NOBO | -9.903 | -9.833 | -9.749 |
| UPSA | -8.115 | -7.942 | -7.768 |

**S1.** Joint model estimates of $\beta_{0}^{[eBird]}$, the scaling intercept to relate eBird abundance estimates from the checklist level to grid cell level.

**S2.** Comparison of precision (i.e., CV[BBS]-CV[joint]; panels A and C) between BBS-only and joint models for relative abundance (*α*; top row) and trend (*β*; bottom row) of black-billed cuckoo. Model-specific estimates of mean relative abundance in are shown in panel B, and estimates of mean trend in panel D. In all panels, blue indicates a positive value/increase and red a negative value or decrease, while 0 is set to white.

**S3.** Comparison of precision (i.e., CV[BBS]-CV[joint]; panels A and C) between BBS-only and joint models for relative abundance (*α*; top row) and trend (*β*; bottom row) of upland sandpiper. Model-specific estimates of mean relative abundance in are shown in panel B, and estimates of mean trend in panel D. In all panels, blue indicates a positive value/increase and red a negative value or decrease, while 0 is set to white.

**S4.** Comparison of precision (i.e., CV[BBS]-CV[joint]; panels A and C) between BBS-only and joint models for relative abundance (*α*; top row) and trend (*β*; bottom row) of black-throated blude warbler. Model-specific estimates of mean relative abundance in are shown in panel B, and estimates of mean trend in panel D. In all panels, blue indicates a positive value/increase and red a negative value or decrease, while 0 is set to white.

**S5.** Comparison of precision (i.e., CV[BBS]-CV[joint]; panels A and C) between BBS-only and joint models for relative abundance (*α*; top row) and trend (*β*; bottom row) of Canada goose. Model-specific estimates of mean relative abundance in are shown in panel B, and estimates of mean trend in panel D. In all panels, blue indicates a positive value/increase and red a negative value or decrease, while 0 is set to white.

**S6.** Improvements in precision (i.e., CV[BBS model] – CV[joint model]) of mean abundance (α) estimates for each grid cell in response to the corresponding distances to edge of the black-billed cuckoo , loggerhead shrike, northern bobwhite, upland sandpiper, black-throated blue warbler, and Canada goose ranges. Distances to the edge of each species’ respective ranges are depicted as 25 percentile ranges. Values above the dashed line indicate that estimates from a joint model were more precise than those from a BBS model, whereas values below that indicate that estimates from a joint model were less precise.

**S7.** Improvements in precision (i.e., CV[BBS model] – CV[joint model]) of trend (*β*) estimates for each grid cell in response to the corresponding distances to edge of the black-billed cuckoo , loggerhead shrike, northern bobwhite, upland sandpiper, black-throated blue warbler, and Canada goose ranges. Distances to the edge of each species’ respective ranges are depicted as 25 percentile ranges. Values above the dashed line indicate that estimates from a joint model were more precise than those from a BBS model, whereas values below that indicate that estimates from a joint model were less precise.

**S8.** Improvements in precision (i.e., CV[BBS model] – CV[joint model]) of mean abundance (α) estimates for each grid cell in response to number of BBS routes surveyed in the corresponding grid cell. Shown are black-billed cuckoo, loggerhead shrike, northern bobwhite, upland sandpiper, black-throated blue warbler, and Canada goose model results. Number of BBS routes are depicted as 25 percentile ranges. Values above the dashed line indicate that estimates from a joint model were more precise than those from a BBS model, whereas values below that indicate that estimates from a joint model were less precise.

**S9.** Improvements in precision (i.e., CV[BBS model] – CV[joint model]) of trend (*β*) estimates for each grid cell in response to number of BBS routes surveyed in the corresponding grid cell. Shown are black-billed cuckoo, loggerhead shrike, northern bobwhite, upland sandpiper, black-throated blue warbler, and Canada goose model results. Number of BBS routes are depicted as 25 percentile ranges. Values above the dashed line indicate that estimates from a joint model were more precise than those from a BBS model, whereas values below that indicate that estimates from a joint model were less precise.

**S10.** Improvements in precision (i.e., CV[BBS model] – CV[joint model]) of mean abundance (α) estimates for each grid cell in response to number of eBird checklists in the corresponding grid cell. Shown are black-billed cuckoo, loggerhead shrike, northern bobwhite, upland sandpiper, black-throated blue warbler, and Canada goose model results. Number of eBird checklists are depicted as 25 percentile ranges. Values above the dashed line indicate that estimates from a joint model were more precise than those from a BBS model, whereas values below that indicate that estimates from a joint model were less precise.

**S11.** Improvements in precision (i.e., CV[BBS model] – CV[joint model]) of trend (*β*) estimates for each grid cell in response to number of eBird checklists in the corresponding grid cell. Shown are black-billed cuckoo, loggerhead shrike, northern bobwhite, upland sandpiper, black-throated blue warbler, and Canada goose model results. Number of eBird checklists are depicted as 25 percentile ranges. Values above the dashed line indicate that estimates from a joint model were more precise than those from a BBS model, whereas values below that indicate that estimates from a joint model were less precise.

**S12.** Number of BBS routes compared to eBird checklists and their correlation to each other for black-billed cuckoo, loggerhead shrike, northern bobwhite, upland sandpiper, black-throated blue warbler, and Canada goose data.

**S13.** Map of North American Bird Conservation Regions (BCRs) from the North American Bird Conservation Initiative^7^. Shaded in red are BCRs 1-7, which contain large areas of temperate or boreal forest or tundra and low densities of people, resulting in fewer count data points from either the North American Breeding Bird Survey or eBird.

**S14.** JAGS code used to estimate mean abundance ($\alpha$), year-specific abundance ($\gamma$), and trend estimates ($\beta$) by analyzing only structured survey (i.e., BBS) data.

**S15.** JAGS code used to estimate mean abundance ($\alpha$), year-specific abundance ($\gamma$), and trend estimates ($\beta$) by jointly analyzing structured survey (i.e., BBS) data and community science (i.e., eBird) data.
